# Development of an ultrasensitive molecularly imprinted poly‐(ortho‐phenylenediamine) based sensor for the determination of melamine adulteration in milk and infant formula

**DOI:** 10.1002/fsn3.2914

**Published:** 2022-05-16

**Authors:** Negin Heydarian‐Dehkordi, Seyyed Siavash Saei‐Dehkordi, Zahra Izadi, Mahdi Ghasemi‐Varnamkhasti

**Affiliations:** ^1^ 185152 Department of Food Hygiene and Quality Control Faculty of Veterinary Medicine Shahrekord University Shahrekord Iran; ^2^ 185152 Department of Mechanical Engineering of Biosystems Faculty of Agriculture Shahrekord University Shahrekord Iran

**Keywords:** electrochemical sensor, melamine, molecularly imprinted polymer, ortho‐phenylenediamine, reduced graphene oxide

## Abstract

A sensitive molecularly imprinted poly‐(ortho‐phenylenediamine) electrochemical sensor was fabricated for selective melamine detection in milk and infant formula. The pencil graphite electrode (PGE) was modified by deposition of Au nanoparticles and reduced graphene oxide (RGO) on its surface. The fabrication of the electrode in various stages was monitored using cyclic voltammetry. The immobilized RGO, MIP, and gold nanoparticles on the PGE surface were morphologically characterized by field‐emission scanning electron microscopy (FESEM). Under the optimized conditions, the linear range and the limit of detection (LOD) were 10^–17^–10^–8^ M and 2.64 × 10^–16^ M (S/N = 3), respectively. The prepared sensor exhibited a good reproducibility and repeatability response. The recovery range of melamine‐spiked milk and infant formula was 92.7%–103.9% and 93.5%–105.8%, respectively. The sensor could apply successfully for melamine determination in milk and infant formula samples.

## Graphical abstract

Design of an AuNPs/MIP/RGO/PGE‐based electrochemical sensor to detect the melamine. Sensor characterization was done by CV, FTIR, and FESEM technique. High reproducibility, sensitivity, and selectivity of MIP electrochemical sensor. Excellent applicability for reliable quantification of melamine in milk samples.

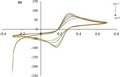

## 1 INTRODUCTION

Milk is an essential nutrient of the human diet that contains carbohydrates, proteins, minerals, and vitamins. The high nutritional value of milk has caused global production and consumption, especially in developing countries (FAOSTAT F, 2016). Milk fraud is a serious concern throughout the world. The more complicated frauds such as adding melamine to increase milk or infant formula nitrogen content have attracted increasing attention from regulators and food researchers. Therefore, applying more sophisticated detection methods is inevitable. As a potential fraud in the milk industry, melamine may be detectable from raw materials to fully processed products. Concerning this fraud, the World Health Organization (WHO) and the US Food and Drug Administration (FDA), as well as China, have set the maximum residue limit (MRL) of 1 mg/kg for melamine in infant formula and 2.5 mg/kg for other products. In Europe, meanwhile, the Food Safety Authority (EFSA) has set a level of 2.5 mg/kg for milk‐based products containing more than 15% milk in their composition. Further, the WHO, EFSA, and FDA considered the tolerable daily intake (TDI) of 0.2, 0.5, and 0.063 mg of melamine per kg of body weight, respectively (Nascimento et al., 2017). Therefore, investigating various toxicological features of melamine‐contaminated foods has received more attention due to their hazardous effects on human health (Nascimento et al., 2017; Vasimalai & John, 2013).

Melamine (1,3,5‐triazine‐2,4,6‐triamine) is a nonedible nitrogen‐rich synthetic molecule (66.6% by weight) that is slightly soluble in water (0.38 or 3.7 g/ml of water at 20 or 90°C, respectively). This white crystalline material has various industrial applications to produce laminates, resins, glues, tableware, adhesives, molding compounds, coatings, plastics, and flame retardants (Daizy et al., 2019; Regasa, Refera Soreta, Femi, & Ramamurthy, 2020). The sources of food contamination with melamine include migrating to food through contact with contaminated instruments, adhesives used in food processing equipment, residues from the use of cyromazine as a pesticide and veterinary drug, as well as the use of trichloromelamine in disinfectant solutions (Grosse et al., 2017).

It is worth mentioning that melamine by itself is slightly toxic. However, when combined with cyanuric acid, it forms insoluble crystals (i.e., melamine cyanurate compound) that can cause tissue damage, including kidney stones, nephrotoxicity, bladder cancer, and even death (Cao et al., 2010; Langman et al., 2009; Lee et al., 2011; Sun et al., 2011).

A wide range of methods such as high‐performance liquid chromatography (HPLC), capillary electrophoresis, and enzyme‐linked immunosorbent assay has been utilized to identify and quantify melamine in various food samples (Daizy et al., 2019). Although these methods have high and variable properties and sensitivities, they are also time‐consuming, expensive, and require highly skilled personnel. Therefore, the development of simple, fast, low‐cost, convenient, and sensitive methods with features consistent with modern world knowledge to detect melamine in milk and dairy products is essential (Daizy et al., 2019).

Molecularly imprinted polymer (MIP) electrochemical sensor is a new device that utilizes molecularly imprinted technique (MIT) and has attracted significant consideration due to excellent applicability to detect various molecules (Kim et al., 2017). This sensor has advantages such as rapidness and specificity for food analysis compared with the conventional analytical instruments mentioned earlier (Ji et al., 2017; Lu et al., 2019). MIPs are synthetic compounds that can predictably identify and selectively interact with a species. The unique properties of MIPs (e.g., desired selectivity, chemical, thermal, and mechanical stability, reusability, ease of preparation, and low cost) make these materials useful for a wide range of applications (Zarejousheghani et al., 2019). Gold and graphene nanoparticles can improve conductivity and electrocatalytic activity, which results in better sensitivity of MIPs (Zhu et al., 2015).

Gold nanoparticles (AuNPs) are suitable nanomaterials to fabricate biosensors due to their large surface area, strong bonding ability, excellent conductivity, and good biocompatibility (Peng et al., 2016). Graphene is a layer whose carbon atoms are arranged in a hexagonal configuration and a two‐dimensional honeycomb lattice. The carbon atoms in graphene are bonded to the SP^2^ hybrid (Nag et al., 2018). Due to its unique properties, such as large surface area, high electrical conductivity, fast heterogeneous electron transfer, and simple preparation, graphene becomes a new promising generation of electrode materials for electrochemical sensors (Nag et al., 2018; Novoselov et al., 2005).

The working electrode plays a fundamental role in the redox process of an electrochemical cell because all the desired reactions occur at its surface. Several types of electrodes like gold, silver, platinum, mercury, glassy carbon, and carbon paste are now used as the working electrodes (Ezhilan et al., 2017). The pencil graphite electrodes (PGE) used to quantify different analytes from a wide range of samples using voltammetric techniques and show reproducible signals with suitable voltammetric peaks. These electrodes can be used as an appropriate tool due to the lack of time‐consuming polishing, reasonable sensitivity, reproducibility, and cost‐effectivity (Purushothama et al., 2018; Sharma et al., 2019). Therefore, in this study, the PGE was selected as the working electrode.

In this work, for the first time an electrochemical sensor based on the electropolymerization of ortho‐phenylenediamine using molecularly imprinted technology to detect melamine was fabricated. This study aims to detect melamine in milk and infant formula using a sensor based on PGE modified with RGO, MIP, and AuNPs using the cyclic voltammetry technique.

## 2 MATERIALS AND METHODS

### 2.1 Reagents and chemicals

Tetrachloroauric (ш) acid trihydrate (HAuCl_4_·3H_2_O), melamine (99%), and potassium ferricyanide/ferrocyanide (K_3_[Fe(CN)_6_])/(K_4_[Fe(CN)_6_]) were purchased from Sigma‐Aldrich. 1,2‐Phenylene‐diamine (C_6_H_8_N_2_), graphite powder, sodium dihydrogen phosphate (NaH_2_PO_4_), sodium hydrogen phosphate (Na_2_HPO_4_), potassium nitrate (KNO_3_), sodium tetrahydroborate, sodium hydroxide (NaOH), potassium chloride (KCl), dimethylformamide (DMF), potassium permanganate (KMnO_4_), hydrochloric acid (HCl), sulfuric acid (H_2_SO_4_), ethanol, methanol, acetic acid, ascorbic acid, l‐histidine, glycine, cyanuric acid, and phenylalanine were bought from Merck. For characterization of modified electrode and investigation of its performance, electrochemical cyclic voltammetry (CV) technique in model solution (5.0 mM Fe(CN)_6_^4‐/3‐^ in 0.1 M potassium chloride) was used. All aqueous solutions were prepared with double‐deionized distilled water.

### 2.2 Equipment

All electrochemical (cyclic voltammetry) measurements were performed using an Ivium potentiostat/galvanostat (Vertex1; Ivium Technologies) run by Ivium software (Eco Chemie) in connection with a three‐electrode system and a personal computer for data processing and recording. A three‐electrode system including an unmodified or modified (RGO/MIP/AuNPs) PGE (with a surface area of 2.1 mm^2^ and 0.7 mm diameter) as a working electrode, an Ag/AgCl (3 M potassium chloride) as a reference electrode, and a platinum wire (with 3 mm length) as a counter electrode was applied. FTIR spectra were recorded and studied in the wavenumber range of 4000–550 cm^−1^ (Thermo Nicolet instrument, Nexus^®^ 670). A Consort pH & EC meter (Model C933) with a combined glass pH electrode was used for pH measurements. Morphological characterization of the MIP‐based electrode was done using a MIRA3 TESCAN field‐emission electron microscope (FESEM).

### 2.3 Preparation of RGO

At the first step conforming to Hummer's method, graphene oxide (GO) was synthesized from pure graphite powder through oxidation in the presence of H_2_SO_4_ and KMnO_4_ (Jin et al., 2018). Because the reduced graphene oxide has better conductivity than the graphene oxide, so 100 ml of the prepared GO suspension (0.25 mg/ml) was sonicated for 30 min. Then, 50 ml of cold distilled water containing 1.25 g of sodium borohydrate (NaBH_4_) was gradually added to GO suspension under ice bath conditions and stirred for 12 h. Finally, it was rinsed three times with distilled water and then dried. To acquire a homogeneous suspension, 5 mg of reduced graphene oxide (RGO) was sonicated with 5 ml of dimethylformamide for 45 min at a temperature of 15°C.

### 2.4 Fabrication of the sensor

First, a bare PGE was cleaned and activated using the CV technique; so that it was placed in 1 M NaOH with the scanning potential of 0.0 to +1.5 V, scan rate of 40 mV/s, and number cycles of 10. The working electrode (length of 3 mm) was immersed in the sonicated RGO solution to immobilize RGO on the treated PGE surface for 60 min. Then to stabilize the RGO, PGE was placed into phosphate buffer solution (0.1 M, pH 7.0), and CV was applied with ten cycles from −0.3 to +0.7 V.

To prepare the MIP solution, the optimal amounts of 0.0126 g of melamine as the template molecule, 0.054 g of ortho‐phenylenediamine as the functional monomer, and 0.058 g of sodium chloride were added to 50 ml of acetate buffer solution (0.5 M, pH 6.0) and was stirred for 15 min to obtain a uniform solution. To form an MIP network on the surface of RGO/PGE electrode, it was placed in the MIP solution and CV technique (the scan rate of 50 mV/s, potential range of −0.2 to +0.8 V, and cycle numbers of 10). Afterward, MIP/RGO/PGE was dried at room temperature and rinsed in deionized water. Finally, AuNPs were formed on the MIP/RGO/PGE electrode by applying a potential of −0.2 V for 150 s in a solution of HAuCl_4_.3H_2_O with an optimal concentration of 0.0005% w/v. After that, it was rinsed in deionized water and dried at room temperature.

### 2.5 Preparation of real sample

The performance of AuNPs/MIP/RGO/PGE to detect melamine in milk and infant formula was evaluated. Briefly, 1 ml of milk or 1 g of infant formula was added to 9 ml of acetonitrile. The samples were sonicated by ultrasonic bath for 10 min and then centrifuged at 8944 × *g* for 10 min. Each sample was passed through a 0.45 μm membrane filter to obtain the supernatant (Ji et al., 2017; Regasa, Soreta, Femi, Ramamurthy, & Subbiahraj, 2020). To obtain the recovery percentages, the supernatant of samples was spiked with different amounts of melamine. Before recovery analyses, the absence of melamine in the real samples was confirmed using the HPLC. Chromatographic analysis was carried out using a Waters 2695 HPLC with a 2478 detector and a C18 column. The mixture of acetonitrile/water (70:30, v/v) was used as the mobile phase. The flow rate and detection wavelength were 1.0 ml/min and 230 nm, respectively.

## 3 RESULTS AND DISCUSSION

### 3.1 Characterization of graphite, GO, and RGO

**FIGURE 1:**
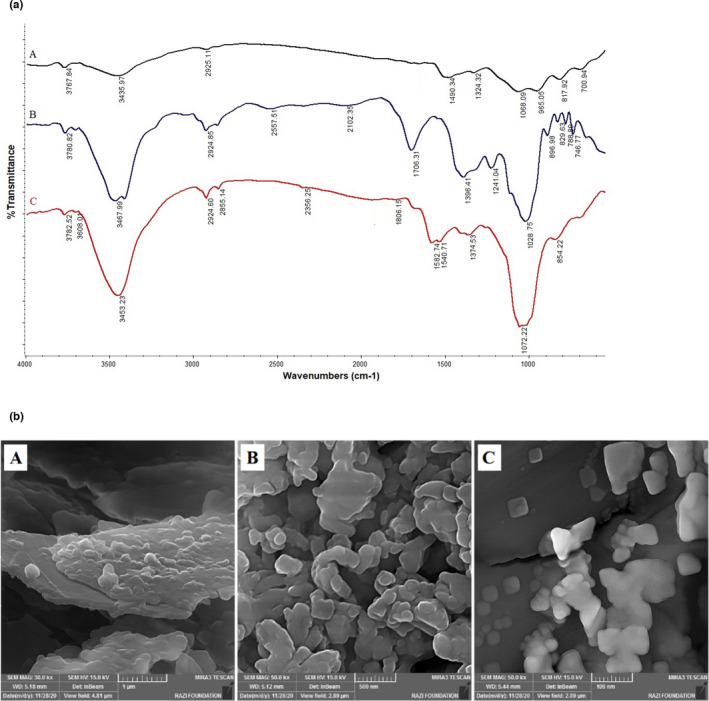
(a) FTIR spectra of graphite (A), GO (B), and RGO (C); (b) FESEM images of the surface of the RGO (A), MIP (B), AuNPs (C)

To confirm the successful synthesis of RGO and investigation of its functional groups, the FTIR analysis was used. In the FTIR spectrum of graphite, no sharp bands can be observed (Figure 1(a)A). However, broadband at 3435 cm^−1^ and two weak absorption bands at 1490 cm^−1^ and 1068 cm^−1^ could be assigned to symmetric stretching vibrations of O–H bonds of graphite oxide impurity and stretching vibrations of C=C and C–O bonds, respectively (Strankowski et al., 2016). After oxidation and synthesis of GO, a broadband at 3467 cm^−1^ and the distinct bands at 1706 and 1028 cm^−1^ could be assigned to stretching vibrations of O–H, C=O, and C–O bonds, respectively. Also, the peaks at 1396 cm^−1^ and 1241 cm^−1^ correspond to bending vibration of carboxylic functional groups and stretching of epoxy C–O, respectively (Anastacio‐López et al., 2019). As can be seen in Figure 1(a)C, all characteristic peaks corresponding to the oxygen‐containing functionalities of RGO have smaller intensities as compared to the GO, and even some, such as peak attributed to the carbonyl groups at 1706 cm^−1^ (Figure 1(a)B), were disappeared in the RGO spectrum. Also, the bands that correspond to the vibration of carboxylic and epoxy on the RGO surface are emerging, which can be confirmed by the successful reduction of graphene oxide to RGO (Emiru & Ayele, 2017).

### 3.2 FESEM characterization

The microscopic evaluations of the structure and morphology of the synthesized RGO and nanocomposite were performed using FESEM techniques. As presented in Figure 1(b)A,B, the synthesized RGO, which has a layered structure, and the amorphous MIP nanostructure formed on the surface can be observed. Also, the immobilized AuNPs with an average size below 100 nm with irregular shapes are shown in the micrograph (Figure 1(b)C).

### 3.3 Electrochemical characterization of the imprinted sensor

**FIGURE 2:**
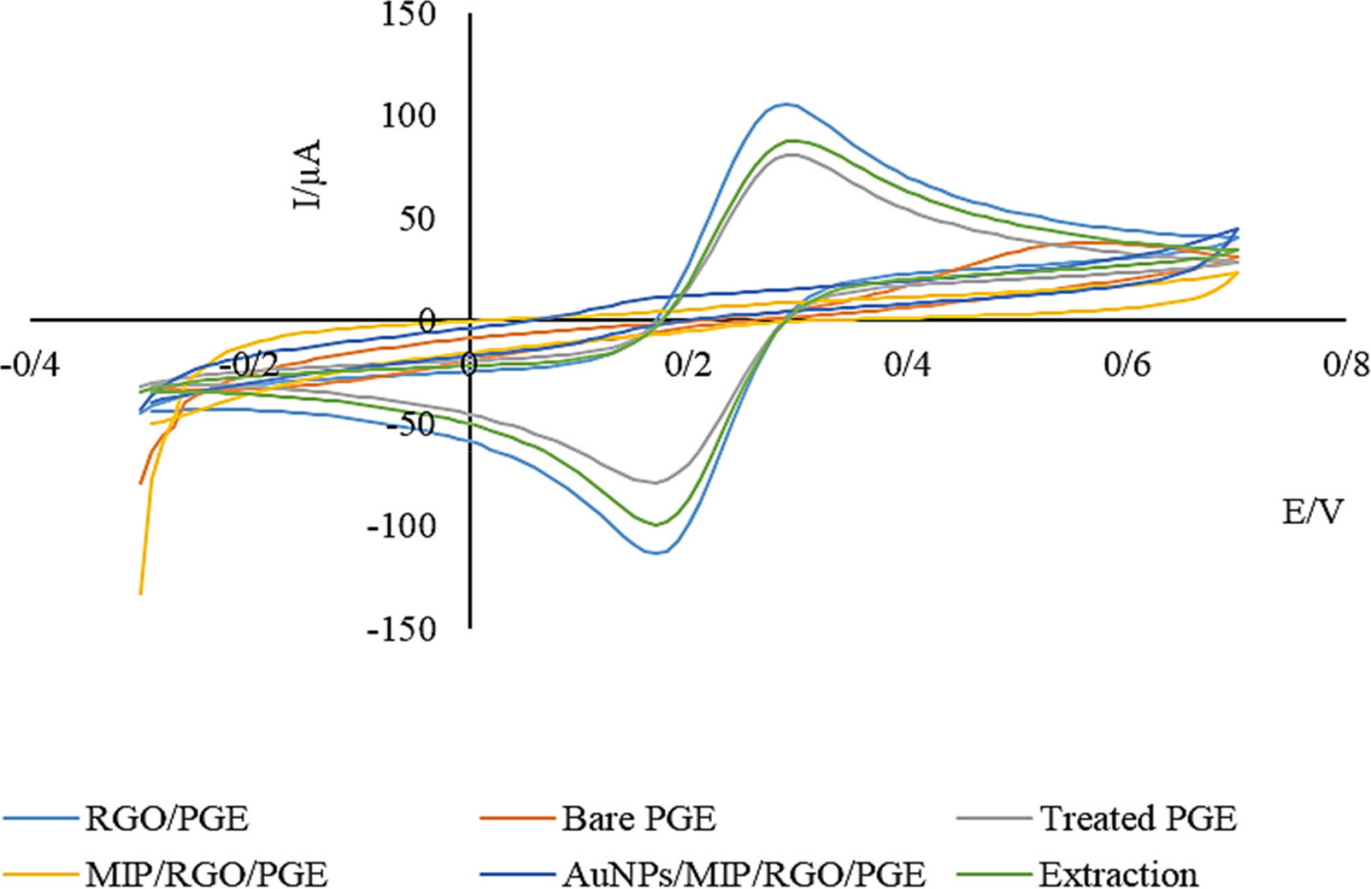
Cyclic voltammograms in the solution of 5 mM [Fe(CN)_6_]^4−/3−^ + 0.1 M KCl at bare PGE, treated PGE, RGO/PGE, MIP/RGO/PGE, AuNPs/MIP/RGO/PGE, and after extraction of template molecule

For stepwise monitoring of electrochemical changes of the sensor during fabrication procedures, CV was employed in 5.0 mM K_3_Fe(CN)_6_/K_4_Fe(CN)_6_ with 0.1 M KCl solution. As shown in Figure 2, the current in the treated PGE curve compared with the bare PGE curve was increased; however, the difference between the reduction and oxidation potentials decreased, which is related to the increase of the electrode surface due to the formation of carbon nanostructures and the oxidation of graphite layers (Özcan & Şahin, 2010). RGO intensified redox current (RGO/PGE curve) by increasing the effective surface area of the electrode and improving the electron transfer kinetics (Mohammad‐Razdari et al., 2019). With the coating of RGO/PGE by dense polymer layer during the electropolymerization process, the current response declined compared to before this process because of probe ions transmission blockage (Figure 2; MIP/RGO/PGE curve). To overcome this problem and expand the electron transfer rate, MIP/RGO/PGE was modified with AuNPs (Figure 2; AuNPs/MIP/RGO/PGE curve). After the extraction of the template molecule from the polymer structure, the redox peak of the probe was increased (Figure 2; extraction curve). In other words, the extraction of the template molecule creates holes and ducts in the polymer network that allow the probe ions to reach the electrode surface and the oxidation‐reduction reaction to occur.

### 3.4 Optimization of experimental conditions

#### 3.4.1 Effect of RGO immobilization time

**FIGURE 3:**
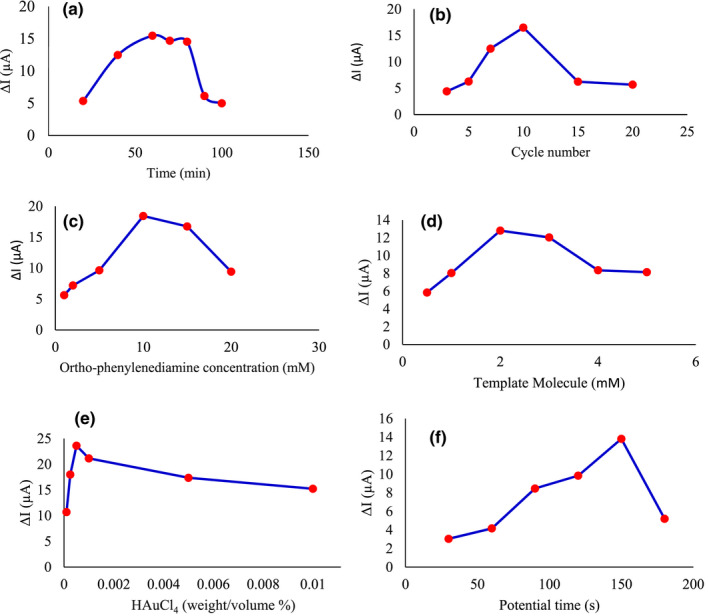
Optimization of impressive parameters on sensor efficiency. Effect of the immobilization time of RGO (a), cycles number of electropolymerization (b), monomer concentration (c), template concentration (d), gold chloride concentration (e), and gold chloride deposition time (f) on AuNPs/MIP/RGO/PGE response

To select the suitable time to immobilize the RGO on the working electrode, the Ip before and after extraction of melamine at different times (20, 40, 60, 70, 80, 90, and 100 min) of immobilization of RGO was obtained. The highest response of the device was observed at 60 min. After this time, it started a downward trend curve, which could be due to the saturation of the electrode surface. According to Figure 3a, 60 min was selected as the optimal time for RGO immobilization.

#### 3.4.2 Effect of the electropolymerization cycles

The number of electropolymerization cycles directly affects the thickness and density of the cavity sizes of MIP film; therefore, it is an important parameter and can affect the sensitivity and linear response of the sensor (Ozkorucuklu et al., 2008; Shamsipur et al., 2018). By comparing the prepared electrodes with a different number of cycles, the number of cycles of 10 was considered as the optimal number of cycles. As shown in Figure 3b, in less and more than 10 cycles, there is a significant reduction in electrode performance. In the number of cycles of <10, a very thin polymer layer is formed on the electrode, and there are no suitable bonding sites in the MIP. Also, the number of cycles of more than 10 has caused the formation of a thick and wide layer of the polymer as well inaccessible sites, and thus the template molecule is not well extracted. Therefore, the next sensors in this study were made under the optimal number of cycles of 10.

#### 3.4.3 Effect of the monomer concentration

The performance of a sensor depends on the concentration of the monomer because the binding ability of the template is related to the number of imprints, and the monomer concentration, in turn, must be proportional to the deposition thickness and the amount of pattern molecule in the polymer matrix (Özcan & Şahin, 2007; Ozkorucuklu et al., 2008). Concentrations (2, 5, 10, 15, and 20 mM) of ortho‐phenylenediamine monomer were investigated, and finally, a concentration of 10 mM was considered as the optimal concentration. As illustrated in Figure 3c, by increasing the monomer concentration up to 10 mM, the sensitivity of the sensor was increased; while at higher concentrations, it results in a larger and thicker polymer layer, which reduces the available diagnostic sites and increases the background current and reduces the response of the device. It is clear that at low concentrations of ortho‐phenylenediamine, a thin polymer layer is formed in which the melamine molecule cannot completely fit in this place and therefore reduce the number of linked sites.

#### 3.4.4 Effect of the template molecule concentration

The concentration of the template molecule has a wonderful effect on the number of bonding sites in the polymer matrix as well as the performance of the sensor. Different concentrations of melamine (0.5, 1, 2, 3, 4, 5 mM) were investigated. As shown in Figure 3d, at concentrations less than 2 mM due to the lack of binding sites, the sensor response decreased. This decrease was also observed at concentrations higher than 2 mM due to molecular aggregation and accumulation. Finally, according to the maximum response of the device, a concentration of 2 mM was selected as the optimal concentration. Based on the results as shown in Figure 3c,d, the optimum molecular ratio of monomer to the template was chosen to be 10^–2^.

#### 3.4.5 Effect of the gold chloride concentration and deposition time

AuNPs are another factor affecting the performance of the electrode, which reduces the background current, increases the electron transfer rate, and increases the device's response. For this purpose, the effect of different concentration of gold chloride (0.0001, 0.00025, 0.0005, 0.001, 0.005, 0.01 weight/volume %) and different deposition times (30, 60, 90, 120, 150, 180 s) on the electrode performance were investigated. As illustrated in Figure 3e, with increasing the concentration of gold chloride up to 0.0005% w/v, the current increases, and for higher concentrations, the current decreases. As a result, the optimal concentration of 0.0005% w/v gold chloride was selected. A time of 150 s was also chosen for deposition of AuNPs (Figure 3f).

#### 3.4.6 Effect of the extracting solvent

**FIGURE 4:**
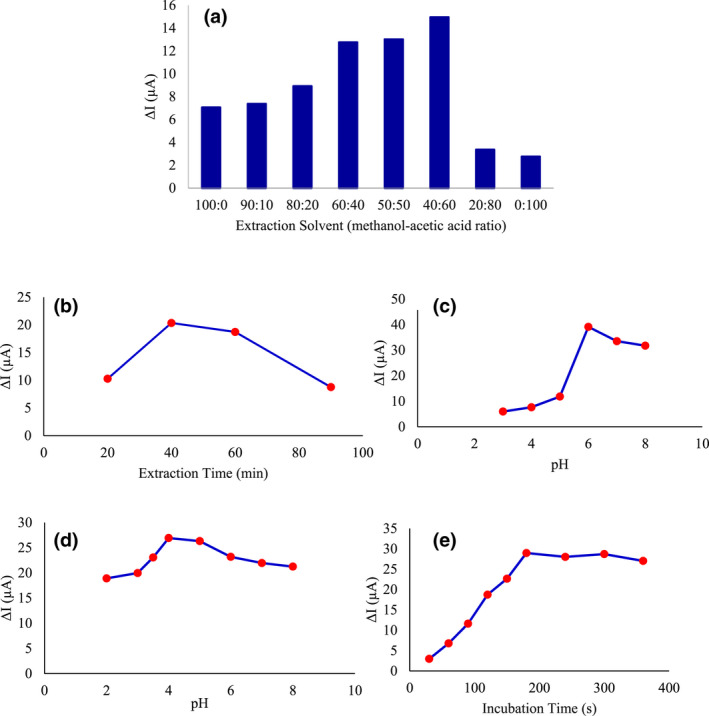
Optimization of effective parameters on sensor performance. Effect of the extracting solvent (methanol‐acetic acid with different ratios) (a), extraction time (b), test solution pH (c), melamine solution pH (d), and incubation time (e) on AuNPs/MIP/RGO/PGE response

Different methods are used to extract the template molecule (Shamsipur et al., 2018), such as redox of the template in the polymer (Liu et al., 2009; Ozkorucuklu et al., 2008), overoxidation of the polymer, (Ozkorucuklu et al., 2008) and supercritical fluid desorption (Ellwanger et al., 2001). In this study, the most common method, i.e., solvent extraction (Shamsipur et al., 2018), was used. In this method, the solvent can interact strongly with the polymer and facilitate the exit of the pattern molecule. For this purpose, acetic acid‐methanol with different ratios (0:100, 10:90, 20:80, 40:60, 50:50, 60:40, 80:20, and 100:0% by volume) were studied. As depicted in Figure 4a, the highest response of the device and, therefore, the best extraction of the template molecule from the polymer structure related to the solvent containing 40% methanol and 60% acetic acid. The removal of the template molecule creates channels in the polymer structure that facilitate the access of K_3_Fe(CN)_6_ to the electrode surface as well as the reduction‐oxidation reaction.

#### 3.4.7 Extraction time effect

After examining this parameter at different times of 20, 40, 60, and 90 min, it was observed that the maximum response of the device was in 40 min (Figure 4b).

#### 3.4.8 Effect of the reaction solution pH

The pH of the test solution is effective on the electron transfer rate, the structure, and the performance of the MIP (Rao et al., 2017). The best state and structure of the analyte in the polymer network can be achieved by optimizing the pH of the reaction solution. For this purpose, molecular imprinted polymer solutions with different pHs of 3, 4, 5, 6, 7, and 8 were investigated. After comparing the cyclic voltammogram of the sensors, the pH of 6 was set as the optimal pH for the MIP solution (Figure 4c).

#### 3.4.9 Effect of the analyte solution pH

The pH of the analyte solution is an important parameter that has a significant effect on the reconnection of the analyte with the MIP (Bakas et al., 2015). The pH of the sample solution plays an important role in the melamine adsorption process by creating both analyte forms in the solution and the adsorbent surface charge (Regasa, Soreta, Femi, Ramamurthy, & Kumar, 2020). Therefore, the effect of different pHs (2, 3, 3.5, 4, 5, 6, 7, and 8) of the sample solution on the amount of melamine uptake by the MIP was investigated. After comparing the cyclic voltammogram sensors, the pH of 4 was considered optimal (Figure 4d). According to the results, it is clear that the sensor response reaches its peak with the increase of the pH up to 4, then follows an almost constant trend in the pH range of 4–5, while it decreases at pH >5.

The reason for the decrease of current pH >4.5 and pH <3 is the presence of melamine as a neutral molecule which mainly shows no current response, and reduction in the amount of protonated melamine ions by converting the melamine to other species such as ammeline and cyanuric acid.

#### 3.4.10 Effect of incubation time in the analyte solution

At this stage, the sensors were placed in the analyte solution with a given concentration (1 × 10^–11^ M) at different times of 30, 60, 90, 120, 150, 180, 240, 300, and 360 s. As expected, melamine restricted the entry of the iron probe through the ducts on the polymer surface and oxidation on the sensor surface by placing it on the electrode surface positions, which resulted in a decrease in the redox current. Figure 4e shows that with increasing incubation time up to 180 s, the response of the device increases, while at times of more than 180 s, the current remains almost constant, indicating the maximum occupation of melamine sites on the electrode surface. The template molecules could produce the functional groups on imprinted cavities that might cause a highly specific capacity to absorb the analyte. Therefore, 180 s was selected as the optimal incubation time to ensure that melamine saturated conditions were achieved.

### 3.5 Method evaluation

The most important parameters in evaluating a method of analysis are selectivity, linearity, repeatability, reproducibility, detection limit, and ability to analyze a real sample. Therefore, in this study, using cyclic voltammetry and under optimal conditions, the mentioned parameters were investigated.

**FIGURE 5:**
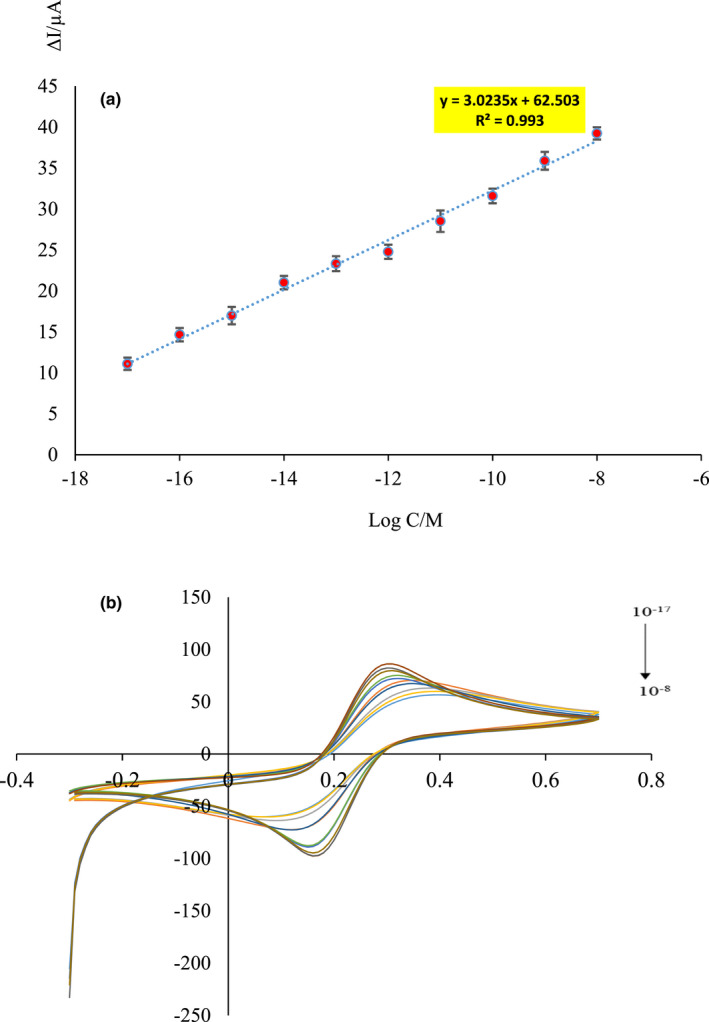
(a) Calibration curve for ΔI versus Log different concentration of melamine. (b) Voltammograms for different concentrations of melamine, 1.0 × 10^–17^, 1.0 × 10^–16^, 1.0 × 10^–15^, 1.0 × 10^–14^, 1.0 × 10^–13^, 1.0 × 10^–12^, 1.0 × 10^–11^, 1.0 × 10^–10^, 1.0 × 10^–9^, and 1.0 × 10^–8^ M, respectively, in the presence of 5.0 mM Fe(CN)_6_^4‐/3‐^ containing 0.1 M KCl solution

The sensor response was recorded as a voltammogram in the presence of Fe probe solution as an electrolyte for each of the melamine concentrations, and the sensor performance was evaluated according to the calibration curve (current vs. concentration, Figure 5a). As the concentration of melamine increases, the MIP sites are occupied, and as a result, the access of K_3_Fe(CN)_6_ to the electrode surface decreases and, consequently, the current decreases (Figure 5b). As it is shown in Figure 5a, the response of the electrode, ΔI (which is obtained from the difference of the current before and after interaction of the electrode with analyte), is linear with Log C in the concentration range from 10^–17^ to 10^–8^ M. The detection limit of the present sensor with a linear equation of *y* = 3.0235*x* + 62.503 and the linearity correlation coefficient of *R* ^2^ = .993 was estimated to be 2.64 × 10^–16^ M, according to LOD = 3*S_b_*/*m*, where *m* and *S_b_* denote the slope of the calibration curve and the standard of the control sample, respectively.

Table 1 shows that the present study had a broader linear concentration range and premier detection limit than previous studies on melamine determination.

**TABLE 1:** Comparison of different MIP sensors for the determination of melamine

Methods	LOD (M)	LR (M)	References
MIP‐electrochemical sensor (goldDMA‐MIP)	1.75 × 10^–12^	10^−11^–10^–4^	Bakas et al. (2015)
MIP‐electrochemical sensor (MI‐PANI‐PIA‐GCE)	1.27 × 10^−13^	0.25–100 × 10^–12^	Regasa, Soreta, Femi, Ramamurthy, and Subbiahraj (2020)
MIP‐electrochemical sensor (MA‐MI‐PANI/GCE)	4.47 × 10^−10^	0.6–16 × 10^−9 M^	Regasa, Soreta, Femi, Ramamurthy, and Kumar (2020)
MIP‐electrochemical sensor (MIP‐MWCNTs/GCE)	5.6 × 10^−13^	10^–12^ ~ 10^–6^	Xu et al. (2018)
MIP‐electrochemical sensor (Au‐MIP)	3.09 × 10^–10^–7.71 × 10^–10^ (complex matrix and PBS solution, respectively)	–	Khlifi et al. (2013)
MIP‐MIL/GCE	8.21 × 10^–12^	10^–11^–10^–6^	An et al. (2019)
MICPS	7.0 × 10^–10^	5.0 × 10^–9^–1.0 × 10^–6^	Li et al. (2011)
MIP‐electrochemical sensor (MIP/AuNPs/RGO/PGE)	2.64 × 10^–16^	10^–17^–10^–8^	This work

Abbreviation: LR, linear range.

To evaluate the sensor repeatability, an electrode was made under optimal conditions, then it was placed in a certain concentration of melamine (1 × 10^–10^ M), and the response of the device was repeatedly recorded five times using the cyclic voltammetry technique. According to the results, the relative standard deviation was 1.51%.

The reproducibility of the sensor was investigated by fabricating eight independent sensors under the same conditions. Then, their current changes before and after being placed in a solution with a certain concentration of melamine (1 × 10^–10^ M) by cyclic voltammetry in a solution of 5 mM ferrocyanide containing 0.1 M potassium chloride were recorded and evaluated. The results showed that the relative standard deviation of current peaks is 4.9%, which indicates the acceptable reproducibility of the sensor.

The stability of the modified electrode is one of the important factors of the performance of a sensor in analytical applications. To evaluate the method stability, three sensors were constructed under optimal device conditions and specifications and then kept in the refrigerator at 4°C. Then every 5 days, the device response was recorded for each sensor and by comparing the results of current peaks compared to the first day. It was observed that the sensor maintained its efficiency of 91.28% on the 20th day. This indicates the acceptable stability of the sensor.

**FIGURE 6:**
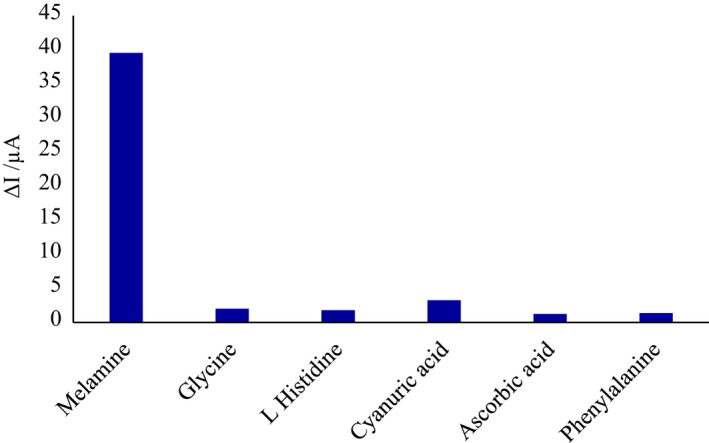
Specificity of the MIP/AuNPs/RGO/PGE with glycine, phenylalanine, l‐histidine, ascorbic acid, and cyanuric acid

For the selectivity test, the specificity of the modified electrode was investigated by comparing the sensor's voltammetric response in the presence of melamine and interfering compounds such as glycine, phenylalanine, l‐histidine, ascorbic acid, and cyanuric acid. According to Figure 6, the response of the sensor to melamine was much higher compared to the mentioned interfering samples, which indicates the ability to uniquely identify the molecular form of polymer compared to melamine molecules. This ability is due to the matching of the functional cavities within the wall, the shape, and the size of melamine during the electropolymerization process.

### 3.6 Real sample analysis

The real sample analysis was carried out by evaluating the MIP‐based electrochemical sensor in milk and infant formula spiked with different concentrations of melamine. The recoveries at three spiked levels are presented in Table 2. The recovery percentage was calculated according to the response signals recorded from the sensor. The range of recovery percentages of 92.7%–103.9% and 93.5%–105.8% were obtained for milk and infant formula, respectively. Vasimalai and John (2013) designed a luminescent sensor to detect the melamine in milk and infant formula. They reported recoveries of 98.87%–100.65% (Vasimalai & John, 2013). Also, Daizy et al. (2019) studied a modified glassy carbon electrode for electrochemical detection of melamine in milk samples. The measured recovery in their study was 98.85% (Daizy et al., 2019). The results showed that the fabricated sensor had a satisfactory recovery to analyze milk and infant formula samples.

**TABLE 2:** Recoveries of spiked melamine into the real sample by the fabricated sensor at ambient conditions

Real sample	Spiked melamine concentration (M)	Determined (M)	Recovery (%)
Milk	1 × 10^–16^	0.9721 × 10^–16^	97.2
	1 × 10^–14^	0.9273 × 10^–14^	92.7
	1 × 10^–9^	1.039 × 10^–9^	103.9
Infant formula	1 × 10^–16^	0.9351 × 10^–16^	93.5
	1 × 10^–14^	1.0582 × 10^–14^	105.8
	1 × 10^–9^	0.9608 × 10^–9^	96.1

## 4 CONCLUSIONS

This study describes an MIP‐based electrochemical sensor for highly sensitive and selective detection of melamine. To the best of our knowledge, this is the first study on an AuNPs‐reduced graphene oxide‐coated electrochemical sensor with the combination of ortho‐phenylenediamine as the functional monomer to detect melamine. Under the optimized conditions, the sensor was efficiently capable of detecting melamine in a concentration range of 10^–17^–10^–8^ with the limit of detection equal to 2.64 × 10^–16^. Overall, the proposed sensor exhibited high sensitivity, selectivity, reproducibility, and excellent recovery values to detect the target melamine in the real samples.

## CONFLICT OF INTEREST

The authors declare that there are no conflicts of interest.

### ETHICAL APPROVAL

This investigation does not involve any human or animal testing.

## Data Availability

The data used to support the findings of this investigation are available from the corresponding author upon reasonable request.
